# Improving Image Super-Resolution Based on Multiscale Generative Adversarial Networks

**DOI:** 10.3390/e24081030

**Published:** 2022-07-26

**Authors:** Cao Yuan, Kaidi Deng, Chen Li, Xueting Zhang, Yaqin Li

**Affiliations:** School of Mathematics and Computer Science, Wuhan Polytechnic University, Wuhan 430024, China; yc@whpu.edu.cn (C.Y.); a1006464380@gmail.com (K.D.); lichen.work@outlook.com (C.L.); deja@whpu.edu.cn (X.Z.)

**Keywords:** deep learning, generative adversarial network, deep generative model, super-resolution, feature transform, multiscale feature extraction

## Abstract

Convolutional neural networks have greatly improved the performance of image super-resolution. However, perceptual networks have problems such as blurred line structures and a lack of high-frequency information when reconstructing image textures. To mitigate these issues, a generative adversarial network based on multiscale asynchronous learning is proposed in this paper, whereby a pyramid structure is employed in the network model to integrate high-frequency information at different scales. Our scheme employs a U-net as a discriminator to focus on the consistency of adjacent pixels in the input image and uses the LPIPS loss for perceptual extreme super-resolution with stronger supervision. Experiments on benchmark datasets and independent datasets Set5, Set14, BSD100, and SunHays80 show that our approach is effective in restoring detailed texture information from low-resolution images.

## 1. Introduction

The main task of image super-resolution (SR) reconstruction is to improve the spatial resolution of low-resolution (LR) images such that the reconstructed high-resolution (HR) image can contain much richer and more detailed textures [1]. The current image SR methods can be divided into two categories: SR reconstruction of a single image and of multiple images [2]. Single-image SR methods collect information from LR data using a specific detector to generate the corresponding HR image. Multi-image SR employs complementary information between the collected LR frames to reconstruct the HR image [3,4,5].

There has been a growing trend in the multiscale representation of images in the last two decades; multiscale representation is now widely used to analyze and model computer vision tasks and has great significance in image applications. Unlike traditional methods, multiscale representation mainly appears in the convolutional neural network as a feature pyramid [6]. Many cross-sectional studies suggest that a feature pyramid could obtain a series of feature maps with different receptive field sizes and scales. A feature pyramid could utilize image context information from local to global perspectives through continuous convolution and down-sampling operations. Fusion of image context information is critical to exploit image context information for image super-resolution because it can effectively increase the accuracy of feature description and enhance the ability of feature representation [7,8].

A generative adversarial network (GAN) [9] provides a robust framework that can generate raw images with high perceptual quality comparable to authentic images. It is now well established that GAN can support image reconstruction through adversarial training, generating more precise and natural HR image textures [10]. However, GAN-based super-resolution reconstruction methods are limited by the current mainstream single-stage scheme that reconstructs images by extracting LR image features followed by up-sampling [11,12]. In comparison, the small size of the LR image may result in high-frequency noise in the reconstructed data. Thus, it is difficult to optimize the network receptive field for texture, and the multiscale context information cannot be fully utilized.

Our scheme proposes an image fusion-based super-resolution reconstruction method that combines multiscale representation and generative adversarial networks. To summarize, our main contributions include the following:

1. We design a pyramid structure generator and expand the original network for multiscale fusion features’ reconstruction.
2. We use a decoder–encoder architecture discriminator and improve the robustness of network training for extreme perceptual super-resolution.
3. We employ an asynchronous network, which can weigh the disparity between the initial training image and the output image at each stage.

## 2. Related Work

**Perceptual Super-Resolution.** Lai et al. [13] proposed a deep Laplacian pyramid network, coined as LapSRN, for fast and accurate super-resolution. LapSRN is inspired by the image pyramid and employs a pyramid network structure. This structure could gradually learn residual mapping for high-frequency components in a coarse-to-fine manner [14]. This technique could significantly reduce the model complexity and the difficulties of learning. Tamar et al. [15] proposed a SinGAN using a single image as input through a fully convolutional GAN pyramid to generate high-quality multi-sample images. SinGAN is suitable for generating single complex natural images. The generated images are diverse and realistic. Tobias [16] et al. proposed a CoSingan trained with a single image at different scales in a multi-stage manner simultaneously. CoSingan provides a way to control the distribution of the internal patch of the training image such that an overall better image structure can be obtained. Haris [17] used a feedforward mechanism to construct a deep back-projection network (DBPN) when processing LR and HR inputs. DBPN emphasizes the SR features using multiple up- and down-sampling stages and could yield promising results on the ×8-fold image super-division.

Motivated by spatial pyramid pooling [18], Zhao et al. [19] proposed a pyramid pooling module to better utilize the global and local contextual information. The low-dimensional feature maps are unsampled to the size of the original feature maps via bilinear interpolation; this module can effectively integrate global and local contextual information. The EDSR-PP model [20] incorporated the pyramid pooling module and further improved the performance over the baseline.

**Extreme Super-Resolution.** In recent years, convolutional neural networks have achieved remarkable success for super-resolution. Dong et al. [21] was the first to use a convolutional neural network for super-resolution reconstruction. This method learns the nonlinear mapping between LR and HR images end-to-end and represents a popular choice for super-resolution reconstruction.

Inspired by deep convolutional neural networks for large-scale image recognition [22], Kim et al. [23] proposed an image super-resolution VDSR reconstruction method with an expanded receptive field by increasing the network’s depth. VDSR can extract more advanced features to alleviate the problem of gradient disappearance. In addition, it can use a greater learning rate for residual learning.

Ledig et al. [24] proposed a practical single-image super-resolution reconstruction method using a generative adversarial network (SRGAN) for more realistic reconstruction. Studies have shown that the use of a discriminator network as an image prior for SR reconstruction can yield better deblurring effects. Wang et al. [25] proposed a spatial feature transform in a generative adversarial network (SFTGAN) and successfully recovered the realistic texture in the reconstructed images. In addition, SFTGAN uses a spatial feature conversion layer to combine classification condition information [26] effectively. Wang et al. [27] also proposed an enhanced SRGAN (ESRGAN) to improve the visual quality and avoid artifacts by introducing residual dense blocks (RRDBs) to train deeper models. Young Hyun et al. [28] doubled the number of RRDBs and used U-net [6] as a discriminator to promote the generator network to produce better super-resolution images.

## 3. Methodology

We propose a GAN-based network that uses a feature pyramid structure for ×16 SR; the whole network comprises a generator and a discriminator. The generator obtains the LR images and transforms them into HR images [29]. The high-resolution feature maps at each stage are entered into the discriminator network for evaluation; the corresponding scores are delivered from the top to the bottom of the tower in the feature pyramid. The discriminator calculates a final score based on the weights of the corresponding stages to determine whether an image is real. After all the stages, the model weight with the highest score is selected to score the image indicators through the specified test set and choose the one that performs better as a result. This cycle continues until the reconstruction satisfies the requirement.

**Figure 1:**
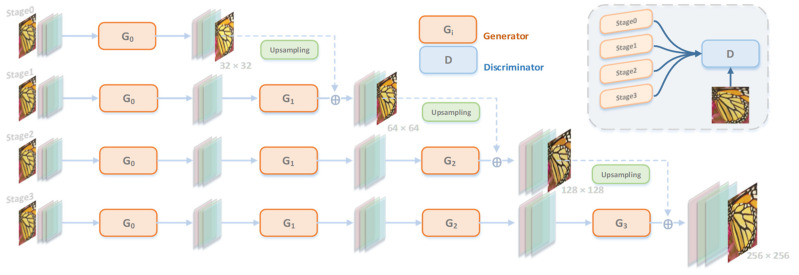
The central network structure diagram of our model. The LR image X is the low-definition image input. X goes to ×16SR across the four stages, generator GW [0,1,2,3], at each time point.

It is important to determine which multiscale pyramid to use. In the original ESRGAN generator network, the size of the images is 250 × 250. When the images are down-sampled more aggressively, the generated images lose their overall consistency because of the insufficient number of layers [18]. We use the progressive multiscale pyramid structure shown in Figure 1 to take the feature information of the LR image as the input, and the forward propagate to three levels. The features are fused after convolution fusion and RRDB residual structure fusion. In the residual module, deep features integrated with well-performing high-frequency information can be found and, at the same time, three different scales are included in the visual map; the number of parameters is reduced by ~50% [30]. The original single training image X and its conditional mask C are initially resized to predefined image scales separately as the training samples $\left\{X_{i}+C_{i}|i\in \left[0,N\right]\right\}$ for the N+1 GANs. The training starts from scale 0 and traverses all image scales. The generator $\left\{G_{0},G_{1},\ldots ,G_{N}\right\}$ in each stage in turn generates a radiological image of a specific scale i, and the output $O_{j-1}$ of $G_{j-1}$ is unsampled to j times image scales, which is further combined with $G_{j}$ to construct the input of $G_{j}\left(j\in \left[1,i\right]\right)$. Because of the previous output $O_{j-1}$ of the generator $G_{j-1}$, $G_{j}$ will not be instantaneously classified as “false” by the discriminator and will continue to fight against $D_{j}$ to gradually learn and generate realistic super-scoring images. Because there is no previous scale, $G_{0}$ will directly map the conditional mask $C_{0}$ to the super-resolution image during the learning process. At this timepoint, the image scale is small and $G_{0}$ will train the result relatively simply and smoothly. The multiscale condition $\{C_{i}|i\in \left[1,N\right]$} resizes the input of the GAN to N times image scales, assuring the output of the GAN matches the given conditions of the input image. The obtained generator can achieve better evaluation in the discriminator [31].

### 3.1. Generator Structure

**Figure 2:**
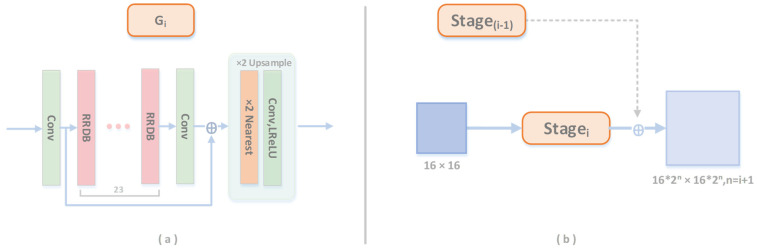
Our generator network structure (**a**). At first, we trained the smallest generator and LR. As the number of stages increases, the capacity and image resolution of the generator will increase (**b**).

Different from ESRGAN [25], our generator network employed a multiscale pyramid structure. Note that ESRGAN removed the batch normalization (BN) layer from SRGAN [24] because BN layers tend to introduce unpleasant artifacts and limit the generalization ability. ESRGAN also replaced the original remaining blocks with RRDBs to improve the performance (Figure 2) because a deeper and more complex network could boost the performance [24]. As shown in Figure 2, we doubled the number of RRDBs and used the residual module to connect the different layer levels.

The training process uses multiple iterations, and each increases the resolution. In the first stage, *G*_0_ fits the input conditions and improves the image resolution at a low scale; the following stages restore the image details, such as low-frequency information that cannot be accurately reconstructed in the previous stage. Therefore, we propose to add the original features’ RRDB residual connection [32] to the output of the newly added convolutional layer and repeat this process *N* times until ×16 resolution is achieved. Under the default settings, we propose to train the last three levels of the generator asynchronously.

In the generator network, the LR images are fed into the entire network. In the first stage, the LR image is up-sampled to the ×2 size by the generator in the initial stage and the feature information is reserved. At each stage, feature information is extracted. This information is eventually combined and passed to the discriminator

**Figure 3:**
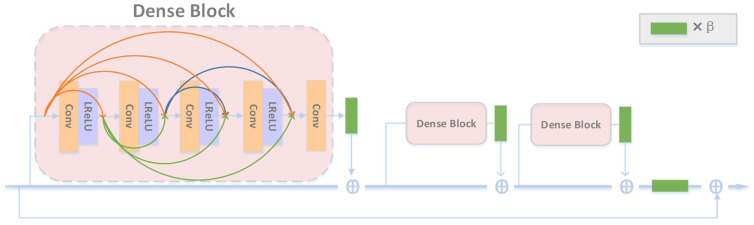
RRDB network structure. β is the scale parameter.

The generator involves in a series of asynchronous training and each stage uses the original features from the previous stage as input (the first few layers are unnecessary). After each stage of training, the images of each level are propagated to the next level. Such layer-by-layer transfer enables learning detailed information based on shallow features. After going through all the stages, the images are up-sampled ×16 and retain the authenticity of high-frequency feature information. As shown in Figure 3, the image and the noise parameters obtained at each stage will be passed to the discrimination network.

As in the original ESRGAN generator network, when the image down-samples more aggressively, the generated image will lose its overall consistency because of insufficient levels. In this way, our method can obtain deeper features in the residual module and fuse feature maps at three different scales to reduce the number of parameters by ~50%. The results in Section 4 show that our scheme yielded better results in terms of evaluation indicators and subjective visual inspection.

### 3.2. Discriminator and Loss Function

**Figure 4:**
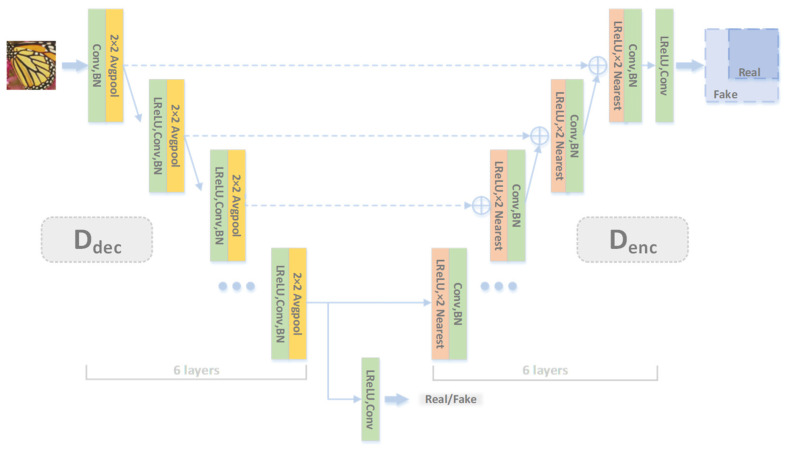
Our discriminator network structure. A U-net structure is adopted to provide feedback for each pixel to the generator [17].

Qualitative methods can be helpful for identifying and characterizing features. Our scheme employed the discriminator’s encoder and decoder. We added a decoder to the ordinary encoder, and this architecture could provide pixel-by-pixel feedback to the generator while maintaining the global contextual information. The features compressed by the discriminator could decide whether or not the input image is real, as shown in Figure 4.

The U-net [33] in Figure 4 maintains global and local data representation and provides more information feedback through pixel-by-pixel transmission. The encoder progressively down-samples the input and captures the global image context like image classification networks. Meanwhile, the decoder performs progressive up-sampling and matches the resolution of the output to the input, enabling precise localization. Skip connections that route data between the two modules at the same resolution could further increase the network’s ability to segment fine details accurately [21]. The discriminator consists of six down-sampling and six up-sampling stages, and the skip connections connect different levels.

The discriminator loss ${ℒ}_{D}$ is calculated at the encoder head $\;{ℒ}_{D_{enc}}$ and the decoder head ${ℒ}_{D_{dec}}$. The discriminator loss formula is hinge loss:

(1) $$\begin{array}{c} {ℒ}_{D_{enc}}=E\left[\underset{i,j}{\sum }\max\left(0,{\left[D_{enc}\left(I^{GT}\right)\right]}_{i,j}\right)\right]+E\left[\underset{i,j}{\sum }\max\left(0,{\left[D_{enc}\left(I^{GT}\right)\right]}_{i,j}+1\right)\right], \end{array}$$

(2) $$\begin{array}{c} {ℒ}_{D_{dec}}=E\left[\underset{i,j}{\sum }\max\left(0,{\left[D_{dec}\left(I^{GT}\right)\right]}_{i,j}\right)\right]+E\left[\underset{i,j}{\sum }\max\left(0,{\left[D_{dec}\left(I^{GT}\right)\right]}_{i,j}+1\right)\right], \end{array}$$

where ${\left[D\left(I\right)\right]}_{i,j}$ is the decision of the discriminator at pixel (i,j). The counter loss of the generator is the average of the losses from the encoder and the decoder.

(3) $${ℒ}_{adv}=E\left[\underset{i,j}{\sum }{\left[D_{enc}\left(I^{Gen}\right)\right]}_{i,j}+\underset{i,j}{\sum }{\left[D_{dec}\left(I^{Gen}\right)\right]}_{i,j}\right].$$

To encourage the generator to focus more on semantic and structural changes, we adopt the consistency regularization used in [17], which obtained CutMix transform [34] to synthesize a new training sample and minimize the loss ${ℒ}_{D_{cons}}$. The total discriminator loss is as follows:

(4) $$\begin{array}{c} {ℒ}_{D}={ℒ}_{D_{enc}}+{ℒ}_{D_{dec}}+{ℒ}_{D_{cons}} \end{array}$$

Traditional image quality metrics (e.g., PSNR and SSIM [35]) tend to generate a higher score for blurred images up-sampled ×16. However, humans prefer translated images. We adapt the LPIPS index [6] as perception loss to mimic the human perception of similarity.

(5) $${ℒ}_{lpips}=\underset{k}{\sum }T^{k}\left(\phi^{k}\left(I^{Gen}\right)-\phi^{k}\left(I^{GT}\right)\right),\;$$

where φ is the feature extractor and τ converts the deep embedding into a scalar LPIPS score. It calculates and averages the score from the *k* layers. The final comprehensive loss in the generator is as follows:

(6) $$\begin{array}{c} {ℒ}_{G}=\lambda_{adv}*{ℒ}_{adv}+\lambda_{lpips}*{ℒ}_{lpips},\; \end{array}$$

where $\lambda_{adv}$ and $\lambda_{lpips}$ are scaling parameters.

## 4. Experiments

### 4.1. Experiment Setup

**Dataset.** Our experiment uses the DIV8K [36] dataset, which contains 800 training images and 100 verification cases, with a maximum resolution of 8 K. The dataset covers various scenes and is designed for ×16 SR or higher specifications. Color blocks of 384 × 384 were randomly cropped from the training images and synthesized to 24 × 24 through bicubic down-sampling. We performed random rotation and left-to-right flipping to increase the size of the training data. We select ten images from the training dataset as the validation dataset.
**Training Details.** An Adam optimizer is used to optimize the network parameters and the learning rate is set to 0.00001 for both the generator and discriminator. Prior to training, we pre-train the model 75,000 times without adding loss. The negative slope of leaky relu is set to 0.2 and 0.1 for the generator and discriminator, respectively. The learning rate $\lambda_{adv}=1\times 10^{-3}$, $\lambda_{fm}=1$, and $\lambda_{lpips}=1\times 10^{-6}$. The model size and the number of parameters for the generator are 127 MByte and 6,681,538, respectively, and these are 77 MByte and 16,808,771, respectively, for the discriminator.

### 4.2. Analysis of Experimental Results

Table 1 compares our method with the following: (i) LILF-EDSR [37]: encoders with up-sampling modules, LILF-RDN: reply on self-supervised SR; (ii) GLEAN [38]: diverse priors encapsulated in a pre-trained GAN. Our method achieves competitive PSNR for the three datasets compared with prior works. Both LILF modules are trained for a specific scale of 18 and they may have more advantages on a specific task than our method. Nevertheless, our approach performed better in the case of ×16.

Several methods have been developed for ×16 SR, but the code is not available. Instead, we compared our network with VGG perceptual loss (Adv + VGG, ${ℒ}_{G}=1E-3,L_{VGG}+L_{pix}$) and without VGG loss. Various models and comparison parameters were used to make the experiment results more objective and persuasive. Specifically, two conventional image quality assessment metrics, PSNR and SSIM (higher is better) [35], were employed and LPIPS was used for perceptual quality evaluation (lower is better). It is well known that PSNR and SSIM prefer blurred images and, in perceptual SR, LPIPS is more highly weighted than PSNR and SSIM. As shown in Table 2, our method achieves the best score. Note that we crop the border by 16 px to avoid boundary artifacts when calculating these metrics.

Based on the model structure, our model is highly scalable in terms of resolution. Therefore, to quantitatively evaluate the effectiveness of the learned continuous representation, besides evaluating the extremely super up-sampling tasks of scales in training distribution, we propose to evaluate normal scales to fit the normal situation. For example, when evaluating scales ×2, ×4, and ×8, we use the low-resolution inputs provided in DIV8K and benchmark datasets (with border-shaving that follows [39]). After preparing datasets at different scales, we enter them into the SRGAN and ESRGAN methods in batches and count the results according to datasets at different scales. Because these two methods use ×2 up-sampling, it is necessary to perform simple processing on the dataset to obtain the results for comparison.

For distribution scales, we observe that our method achieves performance comparable to that of prior works. This is because our models rely on up-sampling, and they are trained with different stages for different scales. The generation scale corresponding to the generator at a specific stage can effectively map the image according to the corresponding distribution. At low-scale magnification, the indicator data of PSNR find it difficult to bridge the gap. Although ESRGAN can generate a realistic image and has a leading edge at the ×2 scale, it is slightly inferior to LILF and our method at larger and extreme scales. The LILF non-distributed approach performs well on continuous scales and scales outside the prediction, and our method outperforms LILF with ×N stages specific training.

**Figure 5:**
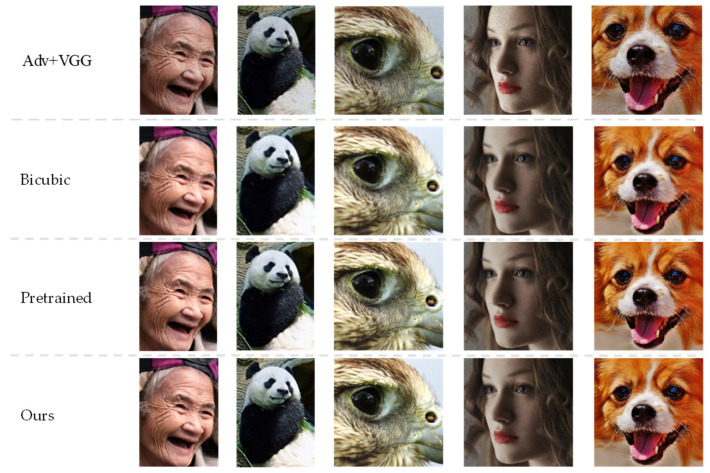
Qualitative results of other methods on the validation set (the images are 112, 420, 448, 575, and 488 from top to bottom). The pre-trained model has limitations in restoring clarity and sometimes produces color artifacts and details inconsistent with the original image. Adv + VGG model produces realistic results while sometimes producing color artifacts and inconsistent details simultaneously. Our model produces results that are more suitable for visual viewing. Please zoom in for a better comparison.

The qualitative results on the DIV8K test dataset are shown in Figure 5. In general, the hair and line’s image quality are slightly improved for LPIPS loss and U-net discriminator. As shown in Table 3, the introduced LPIPS loss could provide better functional space and improve the perceived quality. Meanwhile, the U-net discriminator could provide effective feedback to the generator, covering global and local contexts.

In Figure 5′s pre-trained volume, it can be observed that it is slightly less effective in generating images with more detail. For example, in the parts of the teeth and wrinkles on the image of the old man, the parts of eyes and pupils of the person on the image of the eagle and the woman, and the eyebrows and nose of the person in the fifth group of data, the image recovery of these facial features is not good enough. The proposed model is more advantageous on the validation set, and especially excellent on hair in images of dogs and wrinkles in images of elderly people. The above is a comparison experiment of the validation set, which can clearly show the advantages and disadvantages of each algorithm.

**Figure 6:**
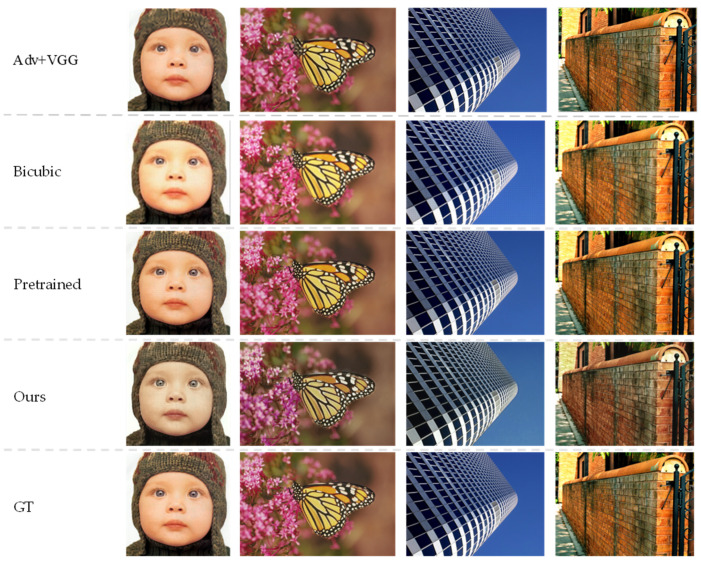
Qualitative results obtained using other methods on the conventional image SR test sets (the images are baby, butterfly, img_005, and img_018 from top to bottom). Adv + VGG model improves the sharpness of the pre-trained model, while our method further enhances the results with consistent details.

More results on public image super-resolution datasets (Set5, Set14, BSDS100, Urban100, and SunHays80) are shown in Table 4 and Figure 6. When ${ℒ}_{lpips}$ only is used without the GAN framework, i.e., ${ℒ}_{adv}$ loss is not considered, the LPIPS [6] score is the best. However, the visual effect is not as good as expected because those repeated pattern artifacts produce inconsistent details. Therefore, although our proposed scheme yielded high LIPPS scores, for some datasets, the visual results can be better. These results suggest that a better LPIPS score does not necessarily indicate better visual quality.

**Figure 7:**
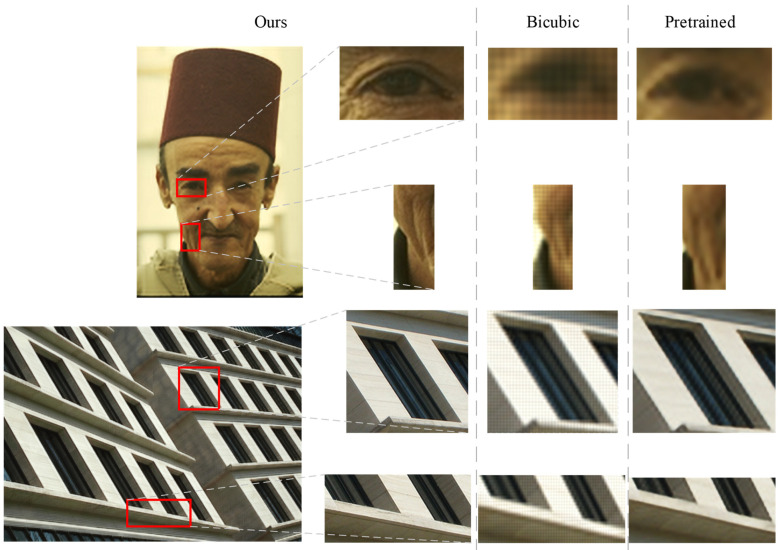
Comparison with typical algorithms: details of the generated images after zooming.

We take a closer look at the features of the baby’s face, the mottled part of the butterfly, the textured part of the building, and the tiled part of the wall in Figure 6. The results of the bicubic algorithm are not clear enough, and the dark parts of the image are rather obvious. Compared with the original image, the edge details shown in the pre-trained model are slightly blurred. The proposed algorithm can generate more iterative details in the image with clear and complete edges than the above algorithm. Figure 7 provides a comparison of the image details under different algorithms. It can be visually seen that our method is clear on the wrinkles of the human face and the straight lines and textures on the rectified buildings. Although there is a slight deviation from the original image, the image portrays a better sense of reality when viewed visually.

In terms of visual impact, the perception loss guides training and allows the network to reconstruct excellent image detail. The lack of texture details on the butterfly image implies that traditional convolutional neural networks are not good at reconstructing images with very rich details. The lack of texture detail on the butterfly image implies that traditional convolutional neural networks are not good at reconstructing images with much detail. The bicubic and pre-trained models lack details and are relatively simple. The proposed algorithm generates an image that deviates slightly from the original image, while the realism of the generated image is better.

More results on public image super-resolution datasets (Set5, Set14, BSDS100, Urban100, and SunHays80) are shown in Table 2 and Figure 6. When *LPIPS* only is used without the GAN framework, i.e., ℒadv loss is not considered, the LPIPS [6] value is the lowest. However, its actual visual effect is not as good as expected, as those repeated pattern artifacts produce inconsistent details. Thus, although some LPIPS values in our proposed scheme are relatively large for specific datasets, better visual results can be seen in our proposed method. The experimental results illustrate that better *LPIPS* values do not always guarantee better visual quality.

## 5. Conclusions

The feature pyramid module we proposed can fully extract and fuse multiscale image features to effectively capture the global context-dependence. In addition, it can complement the original image information using the generated analytical high-frequency information through the transmission of different stages. Comprehensive experiments on various available datasets show that our approach reconstructed images with better visual quality and more details compared with several classical methods.

## Figures and Tables

**Table 1:** Quantitative comparison on ×16SR (PSNR (dB)). Our models outperform other methods in most categories. Best values are shown in bold and second-best values are underlined. When images are non-aligned and contain non-human faces, our models show this as GLEAN. On the Div8K validation set, we achieve a higher similarity than the baseline.

Dataset/Method/PSNR	Bicubic	LILF		GLEAN	Ours
		LILF–EDSR	LILF–RDN		
Cat	16.11	20.85	20.89	20.88	**20.92**
Face	17.27	20.01	20.11	**20.21**	**20.21**
DIV8K	19.47	22.20	22.30	21.30	**22.42**

**Table 2:** Quantitative results of other methods from different scales. Best values are shown in bold and second-best values are underlined. SRGAN and ESRGAN use one model for all scales and are trained with continuous scales uniformly sampled in ×2, ×4, and ×8.

Scales/Method/PSNR	Bicubic	SRGAN	ESRGAN	LILF	Ours (×Nstage)
×2	31.01	34.36	**35.01**	34.67	34.98
×4	26.66	27.02	28.99	29.00	**29.25**
×8	23.54	24.65	25.15	25.23	**26.00**
×16	21.63	21.64	21.76	22.20	**22.42**

**Table 3:** Quantitative results of other methods on the validation set. Better LPIPS value and qualitatively better than the VGG-based perceptual loss shown in Figure 6. Best values are shown in bold and second-best values are underlined.

Method	Bicubic	Adv + VGG	Pretrained	Ours
PSNR	19.47	20.35	20.66	**22.42**
SSIM	0.1122	0.0915	0.1324	**0.1466**
LPIPS	0.8046	0.6532	0.7851	**0.5329**

**Table 4:** Quantitative results with other methods on public image super-resolution datasets. Bolded numbers indicate the best results, and underlined is the second best. Our method shows a better LPIPS value than the others. Although some datasets on PSNR and SSIM are unsatisfactory, they are mainly caused by relatively poor feature perception processing of some image edges.

Method		Bicubic	Adv + VGG	Pretrained	Ours
Set5	PSNR	16.11	17.61	17.59	**17.87**
	SSIM	0.1031	0.1397	**0.1486**	0.1434
	LPIPS	0.5657	0.3625	0.4169	**0.2823**
Set14	PSNR	17.27	18.54	18.36	**18.55**
	SSIM	0.0701	0.0859	0.0802	**0.1072**
	LPIPS	0.6114	0.4504	0.5466	**0.3717**
BSD100	PSNR	17.37	17.94	18.00	**18.22**
	SSIM	0.0604	**0.0992**	0.0691	0.0841
	LPIPS	0.5726	0.5178	0.6887	**0.4112**
SunHays80	PSNR	16.42	16.66	**17.50**	16.68
	SSIM	0.0730	0.0954	0.0934	**0.1026**
	LPIPS	0.7559	0.5450	0.6635	**0.4374**
Urban100	PSNR	16.44	17.04	17.16	**17.43**
	SSIM	0.0636	0.0602	**0.0735**	0.0621
	LPIPS	0.8046	0.8013	0.7986	**0.5780**
